# G-Quadruplexes (GQU)

**DOI:** 10.1155/2018/1079191

**Published:** 2018-04-30

**Authors:** Shozeb Haider, Gary N. Parkinson, Thomas C. Marsh

**Affiliations:** ^1^UCL School of Pharmacy, University College London, London, UK; ^2^Department of Chemistry, University of St. Thomas, St. Paul, MN, USA

The articles in this special edition provide a view of the complexity that guanine bases and their modifications present in both lower and complex organisms and eukaryotic organisms. Guanine nucleotides and guanine-rich nucleic acids have a well-known propensity to self-associate into highly stable structures with a common structural motif of four Hoogsteen H-bonded, coplanar guanines referred to as a G-quartet (aka G-tetrad). A relatively distinctive feature of stacked G-quartets compared to Watson-Crick base paired structures is the presence of a coordination pocket that is often occupied by a variety of physiological cations such as K^+^, NH_4_^+^, Ca^2+^, and Na^+^ as well as others such as Sr^2+^ and Pb^2+^. Overall, the stability and morphology of G-quartet structures are influenced by a combination of intrinsic and external factors (e.g., sequence and coordinating cation) with a growing degree of predictability. Evidence of the broader role G-quadruplexes play in information metabolism has also grown tremendously in the past decades giving us potential therapeutic targets for cancer and similar diseases. Furthermore, the key interactions that guanine makes have been used for their application as electrochemical biosensors and development of nanoscale devices.

Two articles are presented by V. Viglasky et al. In one article, they scan the human and simian immunodeficiency provirus genome for putative G-quadruplex forming sequences. They rationalize that targeting the G-quadruplexes in HIV offers an attractive therapeutic target, which would be of particular use in the development of novel antiviral therapies. The analysis of G-rich regions can provide researchers with a path to find specific targets that could be of interest for specific types of virus. In the other article, they trace the effect of modifications in telomeric sequences in* Tetrahymena* and human repeats. The human telomeric and protozoan telomeric sequences differ only in one purine base (TTAGGG to TTGGGG). They go on to demonstrate that while the substitution does not affect the formation of G-quadruplexes, it does result in an alteration of topology. The results also show that the stability of the substituted derivatives increased in sequences with greater number of substitutions. This observation is somewhat analogous to the phenomenon observed in human telomeric sequences, where the same TTAGGG sequence can adopt six different types of topologies depending upon the chemical environment.

The importance of G-quadruplex function can be emphasized just based on the number of proteins that are being reported that have an association with them. While G-quadruplexes are being touted as potential drug targets and small molecule compounds are being developed, still very little is known about how these multistranded nucleic acids structures interact with proteins. The review by S. A. McKenna et al. focuses on the recognition and comparison of G-quadruplexes by proteins and small peptides, mainly taking into account the X-ray crystallographic and NMR structures, as well as biochemical investigations of binding specificity. These structural features can be used to study and rationally design molecules that target protein-G-quadruplex interactions.

In another article, J. Sagi reviews the structural stability of natural base lesion and synthetic nucleotides. The comprehensive review on the thermodynamic stability of the modified G-quadruplex folds and the stereochemical preferences of more than 70 synthetic and natural derivatives of nucleotides substituting for the natural ones determine the stability and their conformation. The stability of the nucleotide analogs depends on the glycosidic bond conformation, their position of occurrence, and the quadruplex fold. Base modifications hold extreme importance in epigenetic studies. An insight into the thermodynamics of G-quadruplex structural stability can be useful in engineering a stable G-quadruplex topology and in exploring the action of base modifications on G-quadruplex architectures both* in vitro* and* in vivo*.

In another article, G. Wu et al. explore a five-decade old question, what is the handedness of 5′-GMP helical structure? They report, using NMR and IR spectroscopy, the structural details of the helix, which contains 15 nucleotides per 4 turns and only C3'-*endo* sugar puckering. The switch in pH from 8 to 5 results in the helix being devoid of Na^+^ ions, which is in sharp contrast to the 5′-GMP helix formed at pH 8 where the central channel is filled with Na+ ions.

In another article, A. M. Oliveira-Brett et al. review the recent advances in the applications of G-quadruplexes as biosensors. G-quadruplex electrochemical biosensors have received particular attention, since the electrochemical response is particularly sensitive to the DNA structural changes from a single-stranded, double-stranded, or hairpin into a G-quadruplex configuration. The development of an increased number of G-quadruplex aptamers, which combine the G-quadruplex stiffness and self-assembling versatility with the aptamer high specificity of binding to a variety of molecular targets, allowed the construction of biosensors with increased selectivity and sensitivity. The electrochemical characterization, design, and applications of G-quadruplex electrochemical biosensors in the evaluation of metal ions, G-quadruplex ligands, and other small organic molecules, proteins, and cells are reviewed. The electrochemical and atomic force microscopy characterization of G-quadruplexes is presented. Different G-quadruplex electrochemical biosensors design strategies, based on the DNA folding into a G-quadruplex, the use of G-quadruplex aptamers, or the use of G-quadruplex DNAzymes, are discussed.

